# Perceptions of bioenergy with carbon capture and storage in different policy scenarios

**DOI:** 10.1038/s41467-019-08592-5

**Published:** 2019-02-14

**Authors:** Rob Bellamy, Javier Lezaun, James Palmer

**Affiliations:** 1 Department of Geography, University of Manchester, Arthur Lewis Building, Oxford Road, Manchester, M13 9PL UK; 2 Institute for Science, Innovation and Society, University of Oxford, 64 Banbury Road, Oxford, OX2 6PN UK; 3 School of Geographical Sciences, University of Bristol, University Road, Bristol, BS8 1SS UK

## Abstract

There is growing interest in bioenergy with carbon capture and storage (BECCS) as a possible technology for removing CO_2_ from the atmosphere. In the first study of its kind, we investigate whether and how different forms of incentivisation impact on public perceptions of this technology. We develop a new experimental method to triangulate perceptions of BECCS in different policy scenarios through quantitative measurement and qualitative elicitation. Here we show that the type of policy instrument used to incentivise BECCS significantly affects perceptions of the technology itself. While we find approval of coercive and persuasion-based policy scenarios for incentivisation, supportive instruments proved polarising. Payments based on the amount of CO_2_ removed from the atmosphere were approved, but guarantees of higher prices for producers selling energy derived from BECCS were strongly opposed. We conclude that public support for BECCS is inextricably linked to attitudes towards the policies through which it is incentivised.

## Abstract (web summary)

It is not clear how the public views the acceptability of bioenergy with carbon capture and storage (BECCS). Here the authors explored public perceptions of BECCS by situating the technology in three policy scenarios and found that the policy instrument used to incentivise BECCS significantly affects the degree of public support for the technology.

## Introduction

The Paris Agreement on climate change has set out a legally binding commitment to keep the increase in global average temperature to well below 2 °C above pre-industrial levels, with an ambition to limit the rise to 1.5 °C. The Intergovernmental Panel on Climate Change has concluded that meeting these targets is still possible, but nearly all modelling scenarios assume this will require extensive deployment of negative emissions technologies (NETs) by the end of the century, and most of those scenarios have relied on bioenergy with carbon capture (BECCS) to deliver the necessary levels of carbon dioxide removal^1,2^. BECCS involves the generation of energy through the burning of biomass (wood and agricultural products, solid waste, landfill gas and biogas or ethanol and biodiesel) coupled with the capture (via post-combustion, oxyfuel or pre-combustion) and storage of the resulting CO_2_ (CCS) in geological or other long-term reservoirs. If biomass cultivation and associated land-use changes are practised sustainably, this process has the potential to remove CO_2_ from the atmosphere, thereby delivering net-negative emissions.

Despite growing interest in NETs, and in BECCS in particular, it remains unclear how this technology might reshape existing climate policy and energy systems. Indeed, it is currently given a low priority by state and non-state climate policy actors^3^, and progress is increasingly recognised as an uphill struggle rather than the slippery slope that some had anticipated^4^. One particularly understudied question concerns public views on the acceptability of BECCS, and the role that policy instruments might play in shaping those views. This is an urgent question to address, particularly given that BECCS presents significant challenges to dominant energy generation and climate policy regimes^5^, and will not come forward without strong institutional support and significant new incentives for research, development, demonstration and deployment (RDD&D)^6^. In other words, if BECCS is to emerge as a viable option for tackling climate change, it will inevitably do so as an explicitly political technology driven by distinct policy initiatives. Accounting for public preferences and concerns will thus be key to ensuring an effective, acceptable and democratic decision-making process towards the uptake of BECCS, should it be deemed appropriate for support and incentivisation.

This study explored public perceptions of BECCS using an experimental, mixed methods approach that situated the technology within three different policy scenarios (see Methods for full details). Our focus on BECCS had a two-fold purpose. First, despite its high and growing degree of policy relevance, public perceptions of BECCS remain under-researched relative to alternative technologies of greenhouse gas removal and other forms of geoengineering^7–12^. Second, focussing on a single technology allowed a proper investigation of how alternative policy scenarios might affect perceptions of that technology, a question that has largely been ignored in studies of public opinion in this area, and in research on public perceptions of novel technologies more widely. Rather than assuming that public views are formed in relation to the technical characteristics of the technology, we assert that they emerge with regard to tightly coupled socio-technical systems within which those technical features are embedded^11,13–15^. Despite these novel aspects, and in line with increasing moves to broaden research on public participation in the development of new technologies^16^, it is important that our study be seen within the extant wider ecology of studies on the public legitimacy of alternative options for decarbonisation.

To create a study population, a stratified sampling technique was used to recruit a socio-demographically representative and politically diverse cross-section of the public from Oxfordshire, UK. Participants attended a one-day experiment and were divided into three groups, with each being asked to (1) express and discuss their views on BECCS, and (2) deliberate on a different set of policy instruments that could hypothetically be used to incentivise BECCS. To characterise these three alternative BECCS policy scenarios, we used a modified version of Bemelmans-Videc et al.’s^17^ tripartite typology of economic, regulatory and informational policy instruments: carrots, sticks and sermons.

In a coercive policy scenario (group 1), the mechanism of influence on economic operators (specifically fossil fuel energy generating companies) was the threat of removing resources in the event that they failed to effect a transition to BECCS. In a supportive policy scenario (group 2), economic operators were to be influenced by the promise of new resources if they chose to transition to BECCS. Finally, in a persuasive policy scenario (group 3), the actions of economic operators were to be influenced by transferring information and communicating the need for BECCS through reasoned argument. Each of the scenarios was represented in the experiment itself by two concrete policy instruments: taxes and standards in the coercive policy scenario; fixed payments and price guarantees in the supportive policy scenario; lobbying and certification in the persuasion-based policy scenario. While these do not capture the full diversity of policy instruments available to policy-makers in the real world, the scenarios provide a useful heuristic with which to delimit three distinctive and contrasting approaches to the incentivisation of technological transitions.

Here we use this novel experimental approach to analyse perceptions of BECCS in different policy scenarios. First, our study shows a high level of support for BECCS RDD&D, but this is qualified by a range of concerns. Second, we show a high level of support for coercive and persuasion-based policy instruments for incentivising BECCS, particularly in the form of standards and lobbying. Third, we show a statistically significant reduced level of support for BECCS following discussion of the supportive policy scenario. We conclude that the acceptability of BECCS will be determined not merely by its innate technical characteristics, but by the nature of the coupled socio-technical systems within which it is embedded. Furthermore, the salience in our study of a UK-specific policy analogy (price guarantees to energy providers to incentivise nuclear power generation) suggests that public support for BECCS—and possibly for other NETs—will be highly sensitive to features of the national policy context, and specifically to the political acceptability of alternative ways of incentivising the private sector.

## Results

### Perceptions of BECCS at the start of the experiment

Nearly all participants expressed concern about climate change, with 63.6% very concerned and 33.3% somewhat concerned. Similarly, most participants saw the need to tackle climate change as urgent, with 69.7% seeing it as very urgent and 27.3 seeing it as somewhat urgent. However, few participants indicated that they were aware of BECCS prior to the experiment, with 78.8% reporting that they knew nothing or only a little about the subject, and 21.2% reporting that they knew a fair amount.

**Fig. 1:**
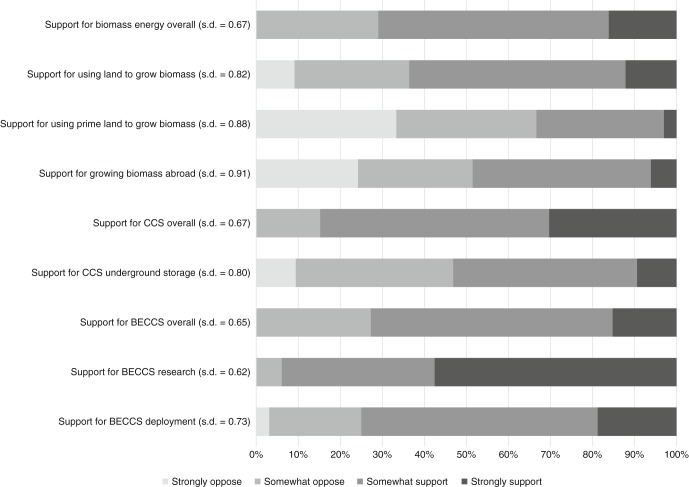
Support for different dimensions of bioenergy with carbon capture and storage at the start of the experiment (% of participants, *n* = 33). Attitudes were elicited on a four-point scale (1 = strongly oppose, 2 = somewhat oppose, 3 = somewhat support, 4 = strongly support). The questions to which each dimension of BECCS pertains can be found in Supplementary Note 1

Fig. 1 shows participant support for different dimensions of BECCS at the start of the experiment. There was more overall support for BECCS than there was opposition, with 72.8% of participants indicating that they were somewhat or strongly in support, and 27.3% somewhat opposed. No participant showed strong opposition. Support for research into BECCS was higher still, with 94.0% somewhat or strongly in support and 6.0% somewhat opposed. Support was slightly lower for deployment, with 75.0% somewhat or strongly in support and 25.0% somewhat or strongly in opposition.

Several concerns qualified support for BECCS. Worries about the geographical footprint of the production networks and other infrastructures that would be required for BECCS consistently emerged in all group discussions. A particular preoccupation was that “we don’t have enough land [in the UK]” to grow sufficient quantities of biomass (group 1, participant I), and that consideration should therefore be given to cultivating biomass in other countries that might be better suited for the task: “You may as well do it in an area that’s ideal for it, rather than trying to do it, cram it into everywhere” (1A). Relatedly, scaling up was a main point of discussion in groups 1 and 2, with participants highlighting that “if it were to be effective the technology has to be generalisable around the planet quite quickly,” and questioning whether it could be “generalised quickly enough for the price [cost] to come down” (2F).

The geopolitical feasibility of pursuing BECCS emerged as an issue in group 1, where some participants mentioned that it might be more feasible in authoritarian countries such as China “because they can just say, all right, you as an individual, too bad, you have to do it, that’s it” (1K). Further to this point, the pursuit of BECCS in developing countries was seen by some as an economic opportunity in that “it’s going to be good for the country because they are going to be exporting the goods [biomass] and getting money back in, and that has to actually help the country as a whole” (1E). On the other hand, ethical concerns were raised in that “it’s as if we’re saying that their land is less important than our land” (1K).

BECCS was not seen as a “one-size fits all solution” across all three groups, with participants instead recognising the need for alternative options (“it makes more sense to say, well in this country this source of renewable energy makes the most sense” (0I)). The question of “why aren’t we focussing more on the renewable sources that are already there” (1A) was a particular concern in group 1’s discussions, raising the possibility that BECCS might be seen as a distraction from those alternatives (a version of the moral hazard or mitigation deterrence argument). Support for research was consistently strong, with some participants in group 1 arguing that this should be driven by business as “it has to be profitable otherwise you aren’t going to do it (1K)”. Similarly, some in group 3 questioned “why should the tax payer pay for it? Because you know, the economy is already pretty shaky without putting through these little ideas. How much is it going to cost?” (3B).

Support for the biomass energy component of BECCS was relatively high, with 71.0% of participants somewhat or strongly in favour and 29.0% somewhat against. No participant indicated strong opposition to biomass energy generation. Support for using land to grow biomass for energy was lower, however, with 63.6% somewhat or strongly in support and 36.4% somewhat or strongly in opposition. Support for using prime agricultural land was lower still, with 33.3% somewhat or strongly in favour and 66.7% somewhat or strongly against. Support for using biomass which has been grown abroad to generate energy in the UK was mixed: 48.5% of participants claimed to be somewhat or strongly in support, while 51.5% were somewhat or strongly opposed.

The potential for land-use conflicts emerged as a concern in all groups with respect to energy generation from biomass, particularly in relation to food production on prime agricultural land. In groups 1 and 2 participants nevertheless suggested several proposals for minimising these conflicts, including arguments stating that there was “no reason why you can’t have agroforestry” (1H) and combine food production and biomass production. Others asked “why can’t we use food waste?” (1A) as a fuel source, or pointed to new technological options to solve some of the bottlenecks (“because we’ve got irrigation, like drip irrigation, we’ve also got like hydroponics, so there are ways to produce a lot more plants” (2A)).

Some participants in groups 1 and 3 felt that transport fuels required to move biomass would likely be fossil-based, and that this “adds emissions to the equation then. If you’re shipping it via diesel steamer then…” (1G). Wider concerns about the environmental impacts that biomass production might have, including the perception in groups 1 and 2 that “at the moment, as far as I’m aware, deforestation outweighs the benefits that can be gained from an increase in biomass” (2L). Other participants suggested that these impacts could be minimised by using “certain [plant] species perhaps that are faster growing that might be able to sustain the need for this biomass” (1G).

There was more overall support for the CCS component of BECCS than there was opposition, with 84.8% somewhat or strongly in support and 15.2% somewhat in opposition. No participant indicated strong opposition. A more focused question on storing carbon in underground geological formations yielded a different picture, however, with 53.1% indicating that they were somewhat or strongly in support, while 46.9% were somewhat or strongly opposed.

In all three groups, discussions about the CCS component of BECCS touched on the safety of storage. A particularly salient worry was leakage of CO_2_ from the storage facility: “shoving CO_2_ underground is fine, but will it leak?” (3I). This worry was lessened where CO_2_ in liquid form was concerned: “The trick is getting the CO_2_ into a liquefied form, which you can then pump from your place of production to the place of storage” (3I). Participants in all three groups compared the storage aspect of CCS with the long-term undertaking and costs associated with radioactive waste disposal: “it’s similar in terms of nuclear energy in that it would be burying the waste in the Earth, and what happens after a certain time?” (1I).

### Policy instrument preferences for incentivising BECCS

**Fig. 2:**
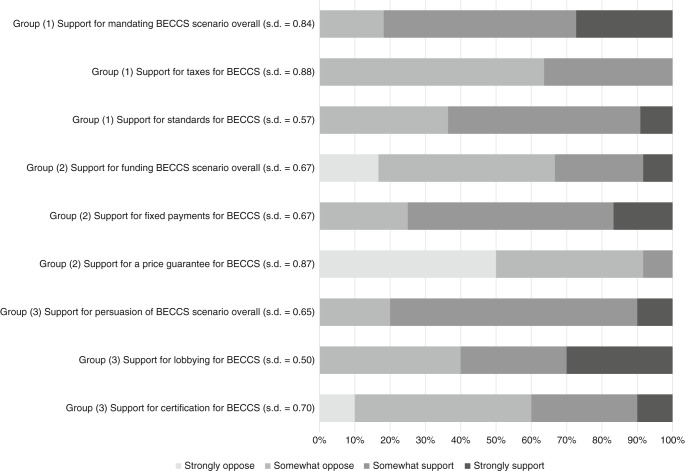
Support for different policy scenarios and instruments for incentivising bioenergy with carbon capture and storage (% of participants, group 1 *n* = 11, group 2 *n* = 12, group 3 *n* = 10). Attitudes were elicited on a four-point scale (1 = strongly oppose, 2 = somewhat oppose, 3 = somewhat support, 4 = strongly support). The questions to which each dimension of BECCS pertains can be found in Supplementary Note 2

Figure 2 shows participant support for different policy scenarios and instruments for incentivising BECCS. The possibility of mandating BECCS through coercive policy instruments received more support than opposition, with 81.8% of the subsection of participants who discussed this option somewhat or strongly in support and 18.2% somewhat in opposition. No participant indicated that they were strongly in opposition. Support for taxes was lower, however, with 36.4% somewhat in support and 63.6% somewhat in opposition. Support for mandatory standards was higher, with 63.6% somewhat or strongly in support and 36.4% somewhat in opposition.

Discussions of the coercive BECCS policy scenario (group 1) focussed on three main issues. The first of these concerned the perceived need for international coordination or some other “super-governmental regulation” (1C) to ensure that national governments did not renege on their commitments to BECCS. The second issue concerned potential resistance from fossil fuel energy companies or even a “full-scale legal blockage” (1A) of such a mandate. The third issue concerned the need for any such mandate to be introduced gradually over a transition period. Reference was made here to the advisability of “going more slowly and encouraging small changes… building it up so the people get used to a change first, and then put on more, rather than putting on 100% of the change all in one go” (1A). Others highlighted the value of building “one or two test sites to feed into the National Grid… and work out all the kinks and issues… and then you could start to expand it to the existing power plants and start determining, can this plant actually be converted? Can this not? Alright, let’s convert the ones that can, and then build others that can” (1K).

New taxes on fossil fuel energy companies in particular were seen as a potential stimulus for secondary industries, providing “the funding and financing for the research for the alternative fuels, without having it going through the energy companies, essentially. And then, through that research, you could create another energy company” (1K). The UK’s plastic bag charge was seen as an analogous, and successful, taxation instrument, being “by far the most effective way at reducing plastic… I don’t think they went far enough with that actually” (1A). On the other hand, these taxes were seen as posing “the risk of falling on the consumer. The company can just put up the price” (1D), resulting in a transfer of cost to consumers. Participants argued that standards should be targeted at the “worst offenders” (1A) and be introduced only after the technology had been developed.

There were much lower levels of support for incentivising BECCS with supportive policy instruments (group 2), with 33.3% of participants somewhat or strongly in favour and 66.7% somewhat or strongly against. Support for fixed payments was high, with 75.0% somewhat or strongly in support and 25.0% somewhat in opposition. Support for a price guarantee was, however, much lower, with only 8.3% somewhat in support and 91.7% somewhat or strongly in opposition.

Discussions of the supportive BECCS policy scenario in group 2 focussed on the potential cost to consumers of new incentives. This was due to the generalised assumption that “energy generated under these conditions will be more expensive than some other types of energy… This would put the energy bills up for sure and therefore a lot of ordinary people… would be against it on cost grounds” (2F).

Fixed payments in particular were praised because they reward companies for “actually taking [CO_2_] out of the air” by the amount removed “rather than just… paying people to have the BECCS technology” (2A), which would not directly incentivise removal and could result in less being removed. This argument was made with the caveat that CO_2_ should be “not only removed, but also properly stored” (2C). A price guarantee, on the other hand, was broadly opposed. This was explicitly related to the use of this policy instrument to subsidise nuclear power generation. Price guarantees were seen as “a big, big problem in relation to new nuclear power stations when they’re guaranteeing very high electricity prices, which they expect us to pay; thank you very much” (2F). The specific example of the nuclear power station currently under construction at Hinkley Point C was raised by participants to argue that price guarantee schemes transfer excessive costs to taxpayers and energy consumers.

There was overall support for the use by governments of persuasive strategies to incentivise BECCS, with 80.0% somewhat or strongly in support of this approach and 20.0% somewhat in opposition. Specific policy instruments fared worse; however, 60.0% of participants were somewhat or strongly in support of lobbying, with 40.0% somewhat in opposition. Support for government-sponsored certification schemes was lower still, with 40.0% somewhat or strongly in support and 60.0% somewhat or strongly in opposition.

Discussions in group 3 focussed primarily on the limited value of persuasion and the need for other forms of incentivisation. There was widespread scepticism about the effectiveness of policies solely based on persuasion and information provision: “personally I think you promote change by regulation, not by persuasion” (3A), “if you’ve got a couple of interested billionaires… maybe you could get a bit of funding to get it off the ground” (3A) and “I would regulate through carbon taxes myself” (3E). Persuasion was seen as potentially a first step before such coercive or supportive measures were applied. Individual persuasion-based instruments were seen as unlikely to succeed in the face of powerful vested interests in the fossil fuel industry. The example of gun ownership laws in America was raised as a relevant analogy: “even with incredibly reasonable evidence, [regarding] America’s gun laws, people [are] getting shot up left, right and centre; still people aren’t really changing their minds about that because of vested interest” (3A).

Certification in particular was seen as needing a negative accreditation component (i.e. “naming and blaming” energy generating companies that failed to incorporate BECCS): “you know we’ve got this Fair Trade certifying… we shouldn’t do it that way round. If you’re Fair Traded you don’t get a label but if you’re not then you get a sticker which, pardon my French, says Bastard Trade” (3D). Certification was also only seen as likely to work if public education on climate change and wider environmental issues were to be significantly increased. Education was seen as being “absolutely central. All right, it’s slow responding, and some people will totally blank it out. And ultimately you do possibly have to have some form of regulation or some sanction at the end of it to justify it” (3D).

### Perceptions of BECCS at the end of the experiment

**Fig. 3:**
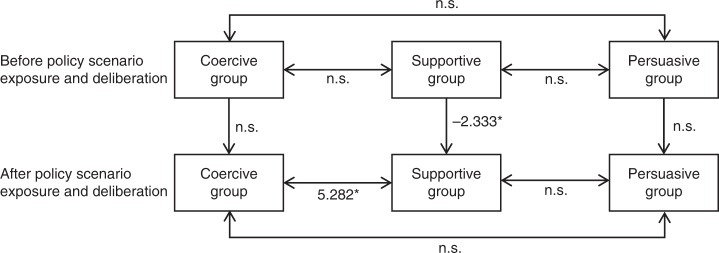
Differences in support for bioenergy with carbon capture and storage between groups before and after exposure to, and deliberation on, policy scenarios (*p < 0.05, n.s. = not significant, *n* = 33). Two-headed arrows indicate statistical tests of difference between the designated different groups, where values provided are the Pearson's *χ*^2^ statistic. Single-headed arrows indicate statistical tests of difference on the designated same group before and after discussion of the policy scenarios, where values provided are the Wilcoxon's signed-rank test *Z* statistic

Following a Shapiro–Wilk test for normality of distribution, a series of non-parametric statistical tests were performed to test (1) the differences between the three groups and (2) the differences between paired samples before and after participation in the discussion of policy scenarios. Figure 3 shows differences in support for BECCS between groups before and after exposure to and deliberation on policy scenarios.

After a non-parametric Levene’s test that verified homoscedasticity in the samples (*p* > 0.05), a Kruskal–Wallis *H* test revealed that before exposure to, and deliberation on, the different policy scenarios, there was no statistically significant difference in the level of support for BECCS across the three groups (*p* > 0.05). A non-parametric Levene’s test then showed that heteroscedasticity arose in the samples after participation in the scenario conditions (*p* < 0.05). A Mood’s median test (which unlike the Kruskal–Wallis *H* test does not assume homogeneity of variance) revealed a statistically significant difference in the level of support for BECCS (*χ*^2^ (2) = 6.199, *p* < 0.05) between the three groups. Pearson's *χ*^2^ and Cramer’s V post-hoc tests revealed that the difference lay between the groups that had engaged with the coercive and supportive policy scenarios for the incentivisation of BECCS (*χ*^2^ (1) = 5.282, *p* < 0.05, *ϕ*_c_ = 0.479). The group that had discussed supportive policy instruments showed a significantly lower level of support for BECCS at the conclusion of the experiment.

A Wilcoxon's signed-rank test revealed that levels of support for BECCS (*Z* = −2.333, *p* < 0.05), its CCS component (*Z* = −3.207, *p* < 0.01) and the underground storage component of CCS (*Z* = −2.333, *p* < 0.05) were all statistically significantly lower after deliberation of supportive policy instruments. In the groups that had deliberated on coercive and persuasion-based policy instruments, there were no statistically significant differences on any questions between their views before and after the discussion.

Consistent with the literature on attitudes towards government intervention in climate change^18^, a Spearman’s *ρ* test revealed a very statistically significant correlation between attitudes towards the role of government and concern about climate change both before (*r*^s^ = −0.457, *p* < 0.01) and after (*r*^s^ = −0.494, *p* < 0.01) exposure to, and deliberation on, the specific policy scenarios, with those opposing government regulation being relatively less concerned about climate change. There was, however, no statistically significant relationship between attitudes towards the role of government and level of support for BECCS.

In sum, those participants who were asked to consider the supportive policy scenario (which included fixed payments and price guarantees) displayed at the conclusion of the experiment a significant reduction in their support for BECCS, while participants in the other groups remained largely constant in their views. Considering that attitudes towards government intervention had no statistical influence on support for BECCS, and that the two policy instruments discussed in the supportive group elicited very different levels of support, it is reasonable to deduce that the reduction in support for BECCS was primarily a result of the extended discussions about price guarantees. Specifically, the reduction in the level of support for BECCS would seem to be the result of the concern that there would be a direct cost to tax payers and energy users if BECCS were to be incentivised by this method.

## Discussion

In this study, we sought to elicit public views on the acceptability of BECCS, and to explore how exposure to specific policy proposals affected initial levels of support for this technology, as one possible approach to carbon dioxide removal.

First, we showed a high level of support for BECCS RDD&D among our participants, despite a generally low level of awareness of BECCS prior to the experiment. This initial position was qualified by a range of concerns, including: geographical footprint, scaling challenges, marginalisation of alternative approaches to climate change, public costs, geopolitical feasibility, land-use conflicts, transport sustainability, environmental impacts, and the safety and duration of carbon storage. Participants also proposed actions for minimising some of these concerns, including: agroforestry, use of food and animal waste as feedstocks, improved irrigation, hydroponics, and faster-growing or higher-yielding crops.

Second, we showed a high level of support among our participants for coercive and persuasion-based policy instruments for incentivising BECCS, particularly in the form of standards and lobbying, but less so from taxes or certification schemes. To be effective, coercive incentives were seen to require global coordination, a gradual transition period, and a commitment to override the expected resistance of fossil fuel energy companies. Persuasion was deemed to be ineffective in the face of vested interests in the absence of additional forms of incentivisation.

Third, we showed a statistically significantly lower level of support for BECCS followed discussion of the supportive policy scenario. In particular, there was a great deal of opposition towards a price guarantee scheme, stemming largely from participants’ knowledge of the high costs being imposed on taxpayers by this mechanism in order to support new nuclear energy provision (i.e. Hinkley Point C). On the other hand, there was a high level of support for fixed payments, which were the highest ranked policy instrument in the study, apparently owing to their ability to establish a direct link between public spending and the climate change performance of BECCS operators.

While our experiment represents one of the first studies into public perceptions of BECCS as a combined approach to tackling climate change, our results are largely consistent with the existing literature on public perceptions of its two components, bioenergy generation and CCS^19–23^. The fact that putting these two components together does not result in a significant shift in attitudes towards the technologies is a notable finding, which extends this literature. Moreover, the combination of bioenergy with CCS does raises a number of novel emergent issues, concerning primarily the intended scale of BECCS deployment. In this way, views on BECCS appear to be influenced by similar considerations to those which apply to other carbon dioxide removal technologies, or even solar geoengineering interventions. For instance, geographical footprint, scaling challenges, marginalisation of mitigation alternatives, and geopolitical feasibility have all featured prominently in studies of public perceptions of these wider technologies^7,9–11^.

Our experiment also shows that the choice of policy instrument with which to incentivise BECCS may have a significant impact on the level of public support for this approach. Those participants in our study who engaged in detail with supportive policy instruments expressed at the end the experiment a significantly lower level of support for BECCS than when they started. This is a reminder that support for BECCS—and indeed for any other proposed NET—is likely to be mediated by public views on the choice of policy used to incentivise it, and not merely by perceptions of climate change or of the particular technical aspects of the approach in question. In other words, the level of public support for a specific technical solution will depend on how it is embedded within broader socio-technical systems and policy frameworks. Further research on public perceptions and the social acceptability of carbon dioxide removal must therefore consider each technological alternative within specific policy and socio-political contexts.

Given that in our study a significant shift in support for BECCS seems to arise from the salience of a UK-specific analogy concerning the incentivisation of nuclear power generation, it will also be crucial for future research to be sensitive to different geographical contexts. Indeed, geographically situated thinking will be vital to understanding how perceptions of emerging socio-technical systems involving BECCS, or other carbon dioxide removal options, are mediated by local contexts and spaces^24^. Correspondingly, any climate policy informed by this research will do well to be consistent with the bottom-up architecture of the Paris Agreement, with incentives being set at the level of state or regional jurisdictions and informed by their preferences of their respective publics, so as to reflect different national political cultures and priorities.

## Methods

### Sample design

We conducted a one-day exploratory experiment in Oxford on 24th March 2018. A stratified sampling technique was used to recruit 33 socio-demographically representative and politically diverse participants from the county of Oxfordshire, UK (see Table 1). The exploratory nature of this research meant that while this was a statistically large sample^25^, this quantitative representation of the target population of Oxfordshire does not extend to broader populations, including the UK at large or indeed to other national contexts. Nevertheless, by sampling for a diversity of political perspectives—both between and within political ideologies (captured by the political party respondents were most likely to support and their attitude towards government regulation of individual behaviours)—we built in an additional, broader level of qualitative representation along those dimensions^26^. The consistency of public perceptions across different countries in relation to other climate engineering technologies^12,27,28^ suggests the possibility of generalising aspects of our results more widely.

**Table 1:** Participants

Sociodemographic variables	Coercive group	Supportive group	Persuasive group	Total
Gender				
Male	4	5	6	15
Female	7	7	4	18
Age				
18–24	3	2	1	6
25–44	5	3	3	11
45–64	3	5	3	11
65+	0	2	3	5
NS-SEC^a^				
1	4	6	5	15
2	3	2	2	7
3	2	1	0	3
4	0	1	2	3
5	2	2	1	5
Political support				
Conservative	4	3	3	10
Green	2	3	2	7
Labour	3	4	2	9
Liberal Democrat	2	2	3	7
Role for government^b^				
More in favour	9	7	6	22
More opposed	2	5	4	11
Participant total	11	12	10	33

^a^UK National Statistics Socio-Economic Classification where occupation and employment status information is used to code five socio-economic class positions in society

^b^Role for government measured the extent to which participants favoured government regulation over individual behaviours. This scale used the short form individualism-communitarianism items by Kahan et al.^31^ and showed a very reliable internal consistency (Cronbach’s *α*  =  0.88)

### Experiment protocol

The one-day experiment comprised several sessions designed to triangulate—through quantitative measurement and qualitative elicitation—public perceptions of BECCS and the effect of different policy scenarios on the level of support for it (see Table 2). The study began with a plenary introduction to climate change (including basic science of observed climate changes, impacts and the policy context of the Paris Agreement) and to BECCS (including a basic overview of its constituent technical components, bioenergy and CCS, and the manner of their combination). Questions were invited and during the subsequent discussions a state-of-the-art technical summary of BECCS’ potentials, costs and risks^29^ was on hand to respond to requests for more information (see Table 3).

**Table 2:** Protocol summary

Time	Activities
09:30–10:00	Participant arrival, registration and completion of consent forms
10:00–10:30	Plenary introduction, presentations on climate change and BECCS and questions invited
10:30–10:45	Allocation to groups and quantitative survey of initial perceptions of BECCS and climate change
10:45–12:00	Facilitated qualitative group deliberation on perceptions of BECCS and climate change
12:00–12:30	Lunch break
12:30–14:30	Facilitated qualitative group deliberation on policy scenarios for BECCS incentivisation
14:30–14:45	Second quantitative survey of perceptions of BECCS and climate change
14:45–15:00	Break
15:00–15:30	Plenary summary of group deliberations and wider participant discussion

**Table 3:** Additional technical information on bioenergy with carbon capture and storage^a^

Technical aspect	Information provided
Carbon removal potential	A median deployment could remove 3.3 Gt C/year (9.5 Gt C were released in 2011, 555 Gt C have been released cumulatively since 1750).
Cost estimate	Medium cost of €100–400/tCO_2_ compared to other NETs.
	Amount of CO_2_ removed would be different in different places.
Vulnerability of stored carbon	Stored carbon would be vulnerable in the long term to climate change, fires, pests, diseases, forestry policy changes.
Capacity to reverse climate change	Could reverse climate change.
Impacts on ecosystems and biodiversity	Large impacts on ecosystems and biodiversity are likely.
Uncertainties	Considerable uncertainties remain over different types of land and biomass, land-use conflicts, impacts on ecosystems, carbon footprint across the supply chain, the readiness of carbon capture and storage, and availability of storage sites.

^a^From European Academies Science Advisory Council report on Negative emission technologies: What role in meeting Paris Agreement targets?

The introduction was immediately followed by a quantitative survey to measure individuals’ baseline perceptions of BECCS (see Supplementary Note 1). The next phase was a 1 h and 15 min facilitated, audio-recorded group discussion designed to elicit qualitative reasonings underpinning those survey responses. Crucially, this phase allowed for participants to introduce and deliberate on their own broader framings of the issue under consideration (which in practice included drawing attention to alternative courses of action for decarbonisation). Facilitators moreover took care not to introduce any framings of their own. The participants were then divided into three homogeneous groups, each designed to be internally heterogeneous socio-demographically and politically. Each subgroup was introduced to a different policy scenario for incentivising BECCS (see section on Policy scenarios). A 2 h facilitated, audio-recorded group discussion then focussed on the respective scenario, paying particular attention to the perceived strengths and weaknesses of the approach and its wider implications for tackling climate change. The experiment concluded with a second quantitative survey designed to measure changes in individuals’ perceptions of BECCS after exposure to the policy scenarios and the subsequent deliberations (see Supplementary Note 2).

Each of the three group discussions was facilitated by one of the authors. Facilitators sought equal contributions by participants, restricting dominant personalities and encouraging talk by quieter participants. Participants were allowed to raise alternative policy scenarios as a means of critiquing the scenario under consideration in their group. If required, facilitators prompted discussion around the strengths and weaknesses of each scenario; the implications of a scenario for citizens; how a scenario might affect energy prices; at what level taxes might be set; when mandates might come into effect; at what level fines may be set; how much governments might pay in fixed amounts; what prices energy sold from BECCS might be set at; whether lobbying would change the behaviour of fossil fuel companies; whether there should be additional prerequisites for earning certification; and what the implications of a particular scenario might be for tackling climate change more broadly.

### Policy scenarios

As outlined in the introduction, three policy scenarios were generated using a modified version of Bemelmans-Videc et al.’s^17^ tripartite typology of economic, regulatory and informational policy instruments^17^. The resulting coercive, supportive and persuasive scenarios were each represented by two concrete policy instruments: taxes and standards in the coercive scenario; fixed payments and price guarantees in the supportive scenario; and lobbying and certification in the persuasion-based scenario. While not exhaustive, the scenarios provide a useful heuristic with which to delimit three distinctive and contrasting approaches to the incentivisation of technological transitions. The scenarios were presented as approaches that are currently being considered by governments around the world (see Table 4).

**Table 4:** Policy scenarios for bioenergy with carbon capture and storage

Policy scenario	Description
Mandating BECCS (group one, coercive scenario)	Mandating BECCS RDD&D would involve governments taking away resources from fossil fuel energy companies or influencing them through rules and directives which mandate them to act in accordance with what is ordered. This would be done through imposing taxes and standards:
	Taxes: Governments would place a carbon tax on new and existing fossil fuel power plants to encourage a shift towards alternative fuels, such as biomass, and the capture of carbon dioxide.
	Standards: Governments would place a direct obligation on new and existing fossil fuel power plants to be converted to biomass energy and equipped with a carbon capture and storage system from a specified date. Failure to comply would result in a fine.
Funding BECCS (group two, supportive scenario)	Funding BECCS RDD&D would involve governments handing out resources to fossil fuel energy companies as well as scientists and entrepreneurs developing BECCS technology. This would be done through providing fixed payments and a price guarantee:
	Fixed payments: Governments would pay a fixed amount to operators of BECCS based on how much carbon dioxide they remove from the atmosphere.
	Price guarantee: Governments would guarantee a higher price for producers selling energy derived from BECCS facilities, as opposed to other kinds of power station.
Persuasion of BECCS (group three, persuasive scenario)	Persuading fossil fuel energy companies to research, develop, demonstrate and deploy BECCS would involve governments transferring knowledge and communicating through reasoned argument. This would be done through lobbying and certification:
	Lobbying: Governments would seek to persuade leaders of the energy sector of the benefits of BECCS as a form of energy generation, and would convene multi-stakeholder fora and industry roundtables to make the case for a transition to BECCS.
	Certification: Governments would introduce an accreditation scheme that would allow companies that produce or distribute BECCS-derived energy to advertise that fact with a special logo.

The first group was confronted with a “mandating BECCS” scenario in which coercive instruments would be used to promote BECCS RDD&D. Based on an analysis of existing policy proposals, this scenario was exemplified with two instruments: taxes (governments would place a carbon tax on new and existing fossil fuel power plants to encourage a shift towards BECCS) and standards (governments would place a direct obligation on new and existing fossil fuel power plants to be converted to biomass energy and equipped with a carbon capture and storage system from a specified date). Failure to comply would result in a fine.

The second group considered a “funding BECCS” policy scenario made up of two supportive instruments: fixed payments (whereby governments would pay a fixed amount to operators of BECCS based on how much carbon dioxide they remove from the atmosphere) and price guarantees (governments would guarantee a higher price for producers selling energy derived from BECCS facilities).

Finally, the third group was invited to discuss a “persuasion for BECCS” scenario organised around two instruments: lobbying (governments would seek to persuade leaders of the energy sector of the benefits of BECCS as a form of energy generation) and certification (governments would introduce an accreditation scheme that would allow companies that produce or distribute BECCS-derived energy to advertise that fact with a special logo).

### Data analysis

The experiment yielded a wealth of quantitative and qualitative data for analysis. Quantitative data were transferred to SPSS statistical analysis software and analysed for descriptive statistics and with non-parametric statistical tests (see Results for more details). Qualitative data were fully transcribed by a professional transcription company and subject to thematic analysis using established procedures for inductive, semantic and constructionist analysis whereby the authors became familiar with the data, generated initial codes, searched for themes, reviewed themes, defined and named themes, and reported them^30.^

### Ethical review statement

The experiment was conducted with ethical approval from the University of Oxford Research Ethics Committee. Informed consent was obtained from all participants prior to their participation.

## Supplementary information

Supplementary Information

## Data Availability

The survey data, audio files and transcripts generated during the current study are not publicly available due to the need to respect participant confidentiality. We will consider requests to make available anonymised survey data and transcripts for research purposes after an embargo period of 3 years, while our research continues.
