# Supplementary material for: Perceptions of bioenergy with carbon capture and storage in different policy scenarios
Source: Nat Commun. 2019 Feb 14;10:743. doi: 10.1038/s41467-019-08592-5 (PMC6375928; doi:10.1038/s41467-019-08592-5)
Supplement: Supplementary file 1 — Supplementary Information [file 41467_2019_8592_MOESM1_ESM.docx]

**Perceptions of bioenergy with carbon capture and storage in different policy scenarios**

Bellamy et al.

**Supplementary Note 1: Initial survey for all participants**

Please write your full name (this will later be anonymised):

People often disagree about climate change and how it should be tackled. Please circle your answer the following questions:

1. How concerned are you about climate change?
   1. Very concerned
   2. Somewhat concerned
   3. Somewhat unconcerned
   4. Very unconcerned
2. How urgent do you think the need to tackle climate change is?
   1. Very urgent
   2. Somewhat urgent
   3. Not particularly urgent
   4. Not at all urgent
3. Do you think that governments are doing enough to tackle climate change?
   1. They are not doing enough
   2. They are doing about the right amount
   3. They are doing too much
4. Overall, to what extent do you support biomass energy?
   1. Strongly support
   2. Tend to support
   3. Tend to oppose
   4. Strongly oppose
5. In general, how supportive are you of using land to grow biomass for energy?
   1. Strongly support
   2. Tend to support
   3. Tend to oppose
   4. Strongly oppose
6. How supportive are you of using prime agricultural land to grow biomass for energy?
   1. Strongly support
   2. Tend to support
   3. Tend to oppose
   4. Strongly oppose
7. How supportive are you of using biomass which has been grown abroad to generate energy in the UK?
   1. Strongly support
   2. Tend to support
   3. Tend to oppose
   4. Strongly oppose
8. Overall, how supportive are you of carbon capture and storage technology?
   1. Strongly support
   2. Tend to support
   3. Tend to oppose
   4. Strongly oppose
9. How supportive are you of storing carbon in underground geological formations?
   1. Strongly support
   2. Tend to support
   3. Tend to oppose
   4. Strongly oppose
10. How much, if anything, would you say that you knew about bioenergy combined with carbon capture and storage (BECCS) before coming to this workshop?
    1. A great deal
    2. A fair amount
    3. A little
    4. Nothing
11. Overall, to what extent would you support BECCS?
    1. Strongly support
    2. Tend to support
    3. Tend to oppose
    4. Strongly oppose
12. To what extent do you support research into BECCS?
    1. Strongly support
    2. Tend to support
    3. Tend to oppose
    4. Strongly oppose
13. To what extent would you support deployment of BECCS?
    1. Strongly support
    2. Tend to support
    3. Tend to oppose
    4. Strongly oppose
14. In general, how do you feel about BECCS?
    1. Very positively
    2. Somewhat positively
    3. Somewhat negatively
    4. Very negatively

People also often disagree about the role of government in society. Please circle the extent to which you agree with the following statements:

1. The government interferes far too much in our everyday lives.
   1. Strongly agree
   2. Moderately agree
   3. Slightly agree
   4. Slightly disagree
   5. Moderately disagree
   6. Strongly disagree
2. Sometimes government needs to make laws that keep people from hurting themselves.
   1. Strongly agree
   2. Moderately agree
   3. Slightly agree
   4. Slightly disagree
   5. Moderately disagree
   6. Strongly disagree
3. It’s not the government’s business to try to protect people from themselves.
   1. Strongly agree
   2. Moderately agree
   3. Slightly agree
   4. Slightly disagree
   5. Moderately disagree
   6. Strongly disagree
4. The government should stop telling people how to live their lives.
   1. Strongly agree
   2. Moderately agree
   3. Slightly agree
   4. Slightly disagree
   5. Moderately disagree
   6. Strongly disagree
5. The government should do more to advance society’s goals, even if that means limiting the freedom and choices of individuals.
   1. Strongly agree
   2. Moderately agree
   3. Slightly agree
   4. Slightly disagree
   5. Moderately disagree
   6. Strongly disagree
6. Government should put limits on the choices individuals can make so they don’t get in the way of what’s good for society.
   1. Strongly agree
   2. Moderately agree
   3. Slightly agree
   4. Slightly disagree
   5. Moderately disagree
   6. Strongly disagree
7. Finally, what is the highest level of science education that you have?
   1. GCSE/O Level/Standard Grades
   2. A-Level/Higher/BTEC
   3. Vocational/NVQ
   4. Degree or equivalent
   5. Postgraduate qualification
   6. Other

**Supplementary Note 2: Second survey for mandating/funding/persuasion of BECCS groups**

Please write your full name (this will later be anonymised):

Please circle your answer the following questions in light of the group discussions around bioenergy with carbon capture and storage (BECCS) being mandated through imposing taxes and standards/ funded through fixed payments and a price guarantee/people being persuaded of bioenergy with carbon capture and storage (BECCS) through lobbying and certification:

1. How concerned are you about climate change after these discussions?
   1. Very concerned
   2. Somewhat concerned
   3. Somewhat unconcerned
   4. Very unconcerned
2. How urgent do you think the need to tackle climate change is after these discussions?
   1. Very urgent
   2. Somewhat urgent
   3. Not particularly urgent
   4. Not at all urgent
3. Do you think that governments are doing enough to tackle climate change after these discussions?
   1. They are not doing enough
   2. They are doing about the right amount
   3. They are doing too much
4. Overall, to what extent do you support biomass energy after these discussions?
   1. Strongly support
   2. Tend to support
   3. Tend to oppose
   4. Strongly oppose
5. In general, how supportive are you of using land to grow biomass for energy after these discussions?
   1. Strongly support
   2. Tend to support
   3. Tend to oppose
   4. Strongly oppose
6. How supportive are you of using prime agricultural land to grow biomass for energy after these discussions?
   1. Strongly support
   2. Tend to support
   3. Tend to oppose
   4. Strongly oppose
7. How supportive are you of using biomass which has been grown abroad to generate energy in the UK after these discussions?
   1. Strongly support
   2. Tend to support
   3. Tend to oppose
   4. Strongly oppose
8. Overall, how supportive are you of carbon capture and storage technology after these discussions?
   1. Strongly support
   2. Tend to support
   3. Tend to oppose
   4. Strongly oppose
9. How supportive are you of storing carbon in underground geological formations after these discussions?
   1. Strongly support
   2. Tend to support
   3. Tend to oppose
   4. Strongly oppose
10. Overall, to what extent would you support bioenergy combined with carbon capture and storage (BECCS) after these discussions, knowing that it would be mandated through taxes and standards/funded through fixed payments and a price guarantee/ persuaded through lobbying and certification?
    1. Strongly support
    2. Tend to support
    3. Tend to oppose
    4. Strongly oppose
11. To what extent do you support research into BECCS after these discussions, knowing that it would be mandated through taxes and standards/funded through fixed payments and a price guarantee/ persuaded through lobbying and certification?
    1. Strongly support
    2. Tend to support
    3. Tend to oppose
    4. Strongly oppose
12. To what extent would you support deployment of BECCS after these discussions, knowing that it would be mandated through taxes and standards/funded through fixed payments and a price guarantee/ persuaded through lobbying and certification?
    1. Strongly support
    2. Tend to support
    3. Tend to oppose
    4. Strongly oppose
13. In general, how do you feel about BECCS after these discussions, knowing that it would be mandated through taxes and standards/funded through fixed payments and a price guarantee/ persuaded through lobbying and certification?
    1. Very positively
    2. Somewhat positively
    3. Somewhat negatively
    4. Very negatively
14. To what extent do you support mandating BECCS through taxes and standards/ funding BECCS through fixed payments and a price guarantee/persuasion of BECCS through lobbying and certification?
    1. Strongly support
    2. Tend to support
    3. Tend to oppose
    4. Strongly oppose
15. To what extent do you support mandating BECCS through taxes/fixed payments/lobbying only?
    1. Strongly support
    2. Tend to support
    3. Tend to oppose
    4. Strongly oppose
16. To what extent do you support mandating BECCS through standards/price guarantee/certification only?
    1. Strongly support
    2. Tend to support
    3. Tend to oppose
    4. Strongly oppose
